# Information Transfer and Criticality in the Ising Model on the Human Connectome

**DOI:** 10.1371/journal.pone.0093616

**Published:** 2014-04-04

**Authors:** Daniele Marinazzo, Mario Pellicoro, Guorong Wu, Leonardo Angelini, Jesús M. Cortés, Sebastiano Stramaglia

**Affiliations:** 1 Faculty of Psychology and Educational Sciences, Department of Data Analysis, Ghent University, Ghent, Belgium; 2 Dipartimento di Fisica, Università degli Studi di Bari and INFN Bari, Bari, Italy; 3 Key Laboratory for NeuroInformation of Ministry of Education, School of Life Science and Technology, University of Electronic Science and Technology of China, Chengdu, China; 4 Ikerbasque, The Basque Foundation for Science, Bilbao, Spain; 5 Biocruces Health Research Institute, Hospital Universitario de Cruces, Barakaldo, Spain; University of Namur, Belgium

## Abstract

We implement the Ising model on a structural connectivity matrix describing the brain at two different resolutions. Tuning the model temperature to its critical value, i.e. at the susceptibility peak, we find a maximal amount of total information transfer between the spin variables. At this point the amount of information that can be redistributed by some nodes reaches a limit and the net dynamics exhibits signature of the law of diminishing marginal returns, a fundamental principle connected to saturated levels of production. Our results extend the recent analysis of dynamical oscillators models on the connectome structure, taking into account lagged and directional influences, focusing only on the nodes that are more prone to became bottlenecks of information. The ratio between the outgoing and the incoming information at each node is related to the the sum of the weights to that node and to the average time between consecutive time flips of spins. The results for the connectome of 66 nodes and for that of 998 nodes are similar, thus suggesting that these properties are scale-independent. Finally, we also find that the brain dynamics at criticality is organized maximally to a rich-club w.r.t. the network of information flows.

## Introduction

Methods based on the theory of complex networks are becoming more and more popular in neuroscience [1]–[3]. Moreover, the inference of the underlying network structure of complex systems [4] from time series data is an important problem that received great attention in the last years, in particular for studies of brain connectivity [2], [5]–[8]. This problem can be handled by estimating from data the flow of information between variables, as measured by the Transfer Entropy (TE) [9], [10] which is a model-free measure designed as the Kullback-Leibler distance of transition probabilities. Recently, in [11], it has been shown that TE is strongly related to Granger causality (GC) [12], [13], a powerful and diffuse model-based approach to reveal (based on prediction) drive-response relationships in dynamical systems: if the prediction error of the first time series is reduced by including measurements from the second one in the linear regression model, then the second time series is said to have a causal influence on the first one.

In a recent paper [14] it was shown that the pattern of information flow among the components of a complex system is the result of the interplay between the topology of the underlying network and the capacity of nodes to handle the incoming information, and that, under suitable conditions, this pattern can reveal the emergence of the law of diminishing marginal returns [15], a fundamental principle of economics which states that when the amount of a variable resource is increased (whilst other resources are kept fixed) the resulting change in the output will eventually diminish. The origin of such behavior resides in the structural constraint related to the fact that each node of the network may handle a limited amount of information. In [14] the information flow pattern of several dynamical models on hierarchical networks has been considered and found to be characterized by an exponential distribution of the incoming information and a fat-tailed distribution of the outgoing information, a clear signature of the law of diminishing marginal returns. This pattern was thus found in artificial hierarchical networks, and in electroencephalography signals recorded on the scalp.

Brain function resides in its ability to process and store information as time goes on. This brain dynamics is best reproduced by neural population models when they are tuned around criticality [16]–[19]. Motivated by this evidence, we planned to investigate how the information generated by a dynamical model flows through a network whose architecture reproduces the structural connections of the human brain. The Ising model displays a remarkably rich dynamics given its simple form, and some of its properties have already been studied on network structures [20]–[24]. We here implement Ising model with Glauber dynamics [25] on weighted, symmetrical connectivity graphs obtained by structural measurements of the human brain and estimated numerically the information transfer between spins.

Varying the temperature, the system susceptibility shows a peak which is related to a phase transition occurring in the limit of large networks [26], and thus characterized by long range correlations; although we are dealing with a network of finite size, we will refer to the temperature at the susceptibility peak as the *critical* temperature of the system. At two different scales, we have found that at criticality the Ising model dynamics results in the maximal amount of total information transfer among variables and that this information transfer is affected by the law of diminishing marginal returns, as it can be seen by comparing the distributions of incoming and outgoing information. The spatial modulation of this phenomenon is analyzed by evaluating, at each node, the ratio 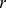 between the outgoing and the incoming information. It turns out that 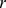 is related to the in-strength of the network nodes: nodes with high 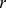 are those more prone to become bottlenecks as the information flow increases. We also characterize the critical state in terms of the average time between spin flips, thus putting in evidence the regions which are most involved in the phase transition. Moreover we also look at how the hubs of the information flow network among spins are connected between them, as the temperature is varied: hubs are more likely to be connected to other hubs than to other nodes (rich club structure) at criticality.

## Results

**Figure 1:**
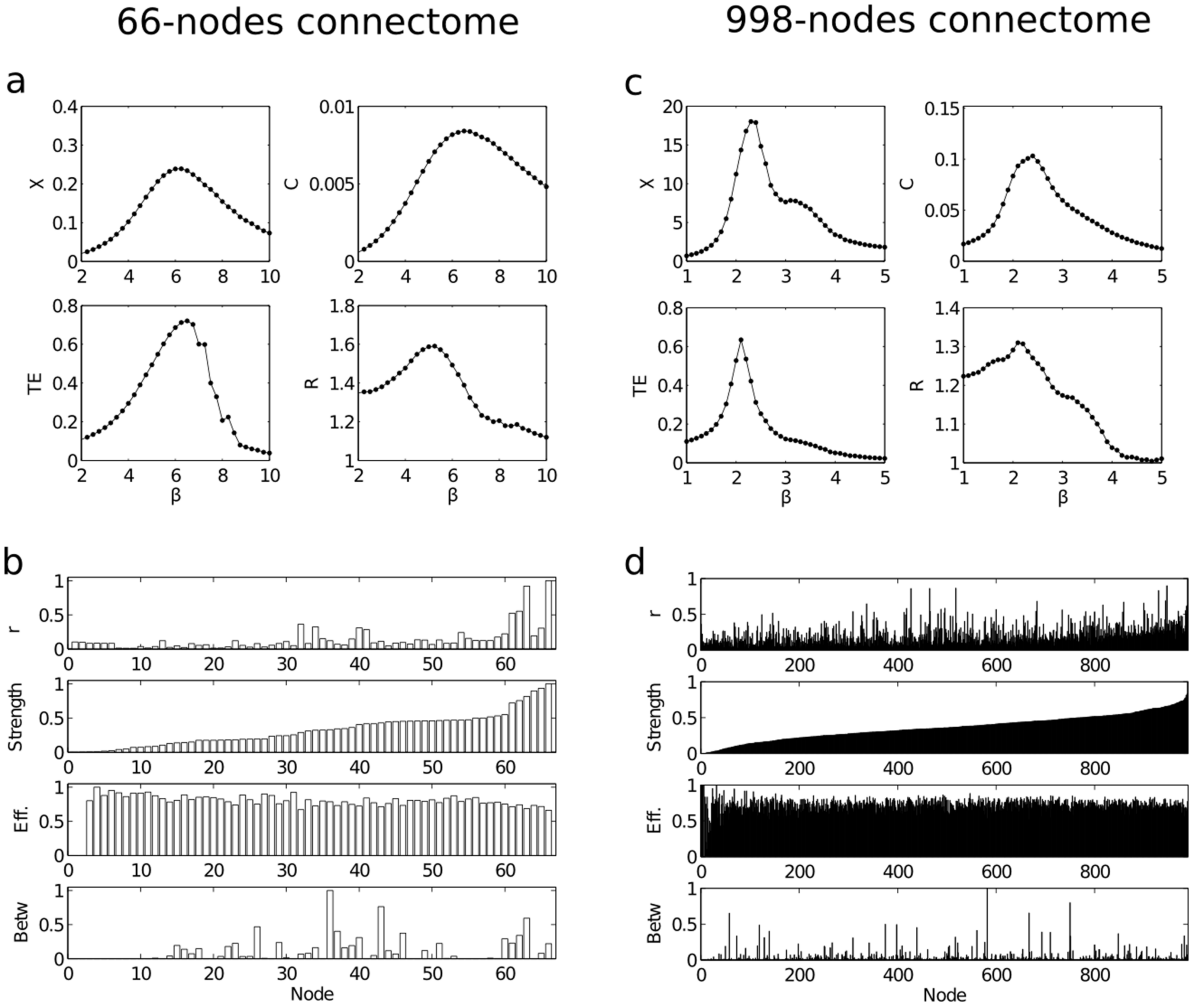
Ising model on the different brain connectomes. a,b: 66-nodes connectome. c,d: 998-nodes connectome. a: The following quantities are depicted versus the inverse temperature 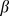: the susceptibility 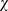; the heat capacity C; the total transfer entropy TE, i.e. the sum of all the information flows in all network pairs; R the ratio between the standard deviations of outgoing and incoming information flows). b: The distribution of the node-measure 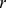 in comparison with different topological measures such as the nodes strength (the sum of the connections weights to each node in the connectivity matrix 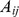), the efficiency and the nodes betweenness. The values of r have been normalized to the interval [0,1]. Nodes have been ordered according to increasing strength. Note that r is not fully explained by the strength as there are nodes with intermediate strength but with high r. Here the value of the temperature corresponds to the one that maximized R but similar patterns are obtained varying 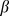. c: same as in panel a but for the 998-nodes connectome. d: same as in panel c but for the 998-nodes connectome.

Figure (1) refers to results of simulating the Ising model on the brain network at different mesoscopic scale, 66-nodes connectome and 998-nodes connectome. Figure (1a) is showing for the 66-nodes connectome four quantities as a function of the inverse temperature 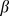 of the Ising model: (1) the susceptibility 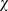, whose peak corresponds to criticality (pseudo-transition); (2) the heat capacity 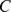, which also provides a signature of criticality (3) the total information flow (the sum of the 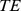 in all network pairs) and (4) the ratio between the standard deviation of the distributions of the outgoing information and the incoming information [7], 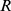. For symmetric interactions, the network of information flows should be also symmetric (i.e., 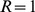), unless some units have a finite capacity close to saturation: in such case the distribution of incoming information is sharper than the one of outgoing information. This is exactly why R is an indicator of the law of diminishing marginal returns [14]. Notice also that R is a quantity calculated in this case at a global level, pooling all the nodes together. We find that around this critical state of the Ising model the total amount of information transfer and 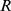 assume large values; while the total transferred information is maximum at the critical point, 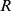 is maximized for an higher temperature. At criticality some units are close to be receiving the maximal amount of input information. As the temperature approaches the critical value, both the input and the output information grow, but their ratio is maximal in the paramagnetic phase. The peak of 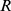 approaches the peak of the susceptibility for larger networks, suggesting a finite size effect explanation for this phenomenon.

**Figure 2:**
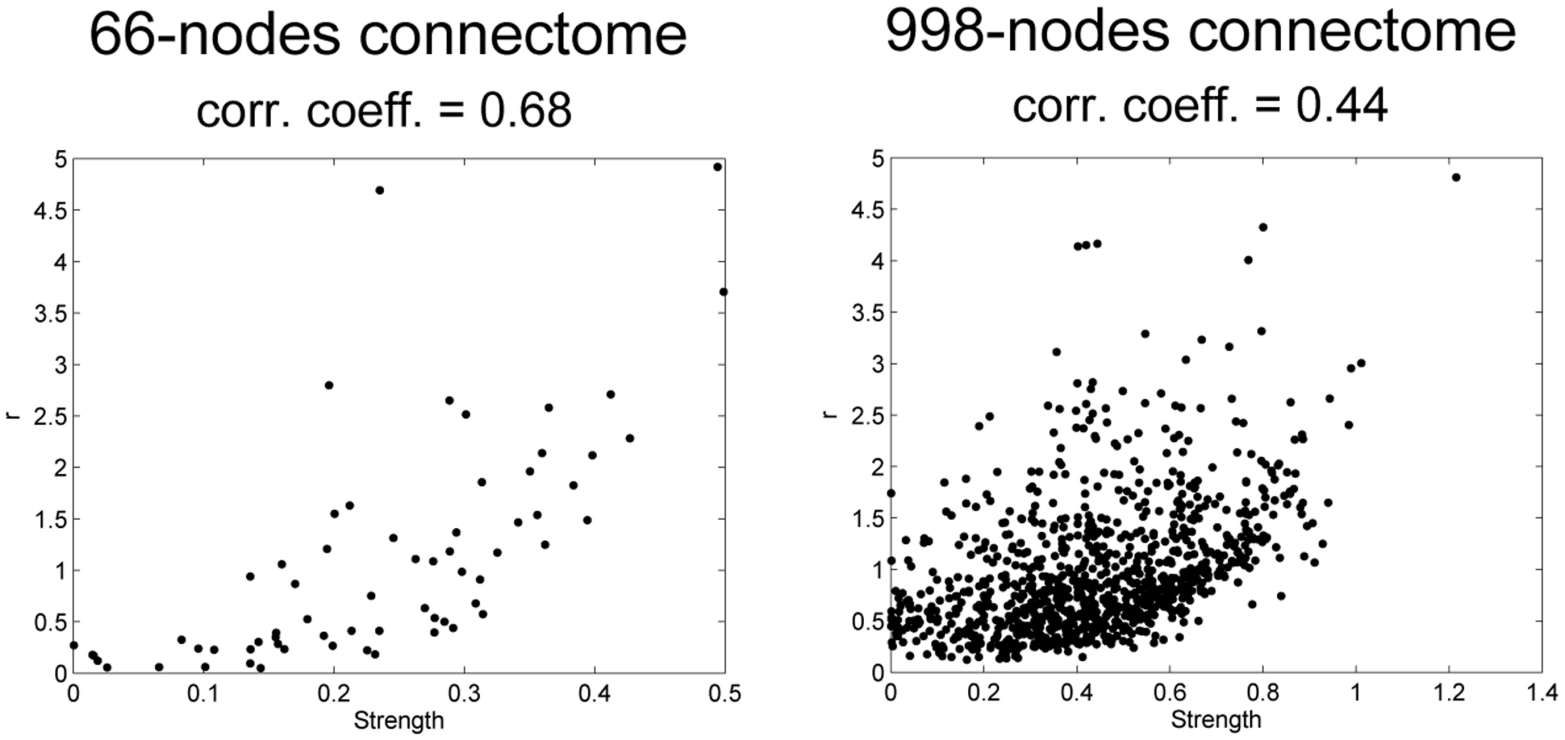
Ratio r of outgoing and incoming information per node at criticality. r is depicted as a function of the nodes strength for both 66 and 989 nodes connectome.

The local modulation of the law of diminishing marginal returns can be analyzed evaluating at each node the ratio between the outgoing and the incoming information 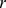 (in contrast with R which is a global network measure). Figure (1b) refers to the value of 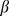 leading to the maximum of 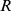, and describes 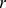 compared with topological properties of the graph, such as the strength, the node-efficiency and the node-betweenness; nodes have been ordered according to growing values of the in-strength, i.e. the number of incoming links. Thus, this is showing that other network properties related with “hubness” such as efficiency (connected with the shortest path length between neighbors of a given node) and betweenness (accounting for the number of shortest paths passing through a given node) are not correlated with the the r value in each node. In [14] it was shown that 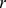 is correlated with the degree for three models of dynamical networks: here we show for the first time that a similar relation holds for the Ising model on the connectome. Indeed, we observed that 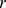 is to some extent correlated to the strength and it thus reflects an intrinsic property of nodes, the propensity to become bottlenecks of information (Figure 2, Pearson correlation coefficient of 0.68). There is no correlation with other network properties (Pearson coefficients equal to 0.05 with Efficiency and 0.04 with Betweenness Centrality). Turning to the ratio S between the intra-hemisphere information flow and the inter-hemispheres information flow, measuring the *segregation* of the network, we find that as the temperature is lowered (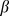 is increased), the hemispheres become more and more segregated.

Next, we asked whether the results found in the 66-nodes connectome can be extrapolated to the 998-nodes connectome. Similar to figure (1a), figure (1b) is showing the quantities 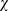, 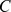, 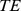 and 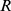 but for the 998 nodes anatomical network. The emerging patterns are similar to those corresponding to the 66 nodes connectome, but in this case 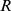 is also maximal close to criticality. Similarities between figures (1b) and (1d) were also found. However, when increasing the spatial resolution, the parameter 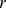 is now less related to the strength (Figure (2), with Pearson correlation coefficient of 0.44. The correlation with other network measures remain close to zero (Pearson coefficients equal to −0.02 with Efficiency and 0.01 with Betweenness Centrality).

**Figure 3:**
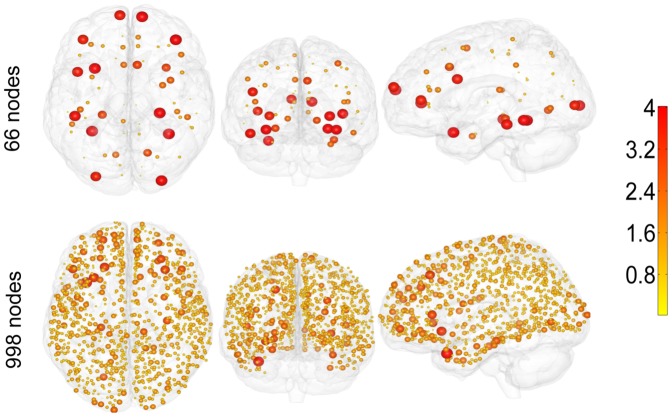
Localization of areas which are bottlenecks of information. The value of r for each region for the Ising model on brain networks, for the 66-nodes connectome (top) and the 998 nodes one (bottom). The size of the spheres is proportional to r, thus showing the most prone regions to became bottlenecks of information.

We have found some regions as potential bottlenecks; all of these regions are symmetrical in the two hemispheres: Superior Frontal Cortex, Precuneus, Superior Temporal Cortex, Medial and Lateral Orbitofrontal Cortex, see figure (3) where the modulation of 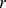 over the brain is shown. Some of these regions are considered as hubs both for the structural and for the functional connectome [27]. It is worth recalling anyway that being a hub (in particular for incoming connections) does not necessary imply that a node is a bottleneck of information flow. The modulation of 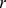 over the brain for the 998-nodes connectome appears to be consistent with the pattern corresponding to 66-nodes connectome.

**Figure 4:**
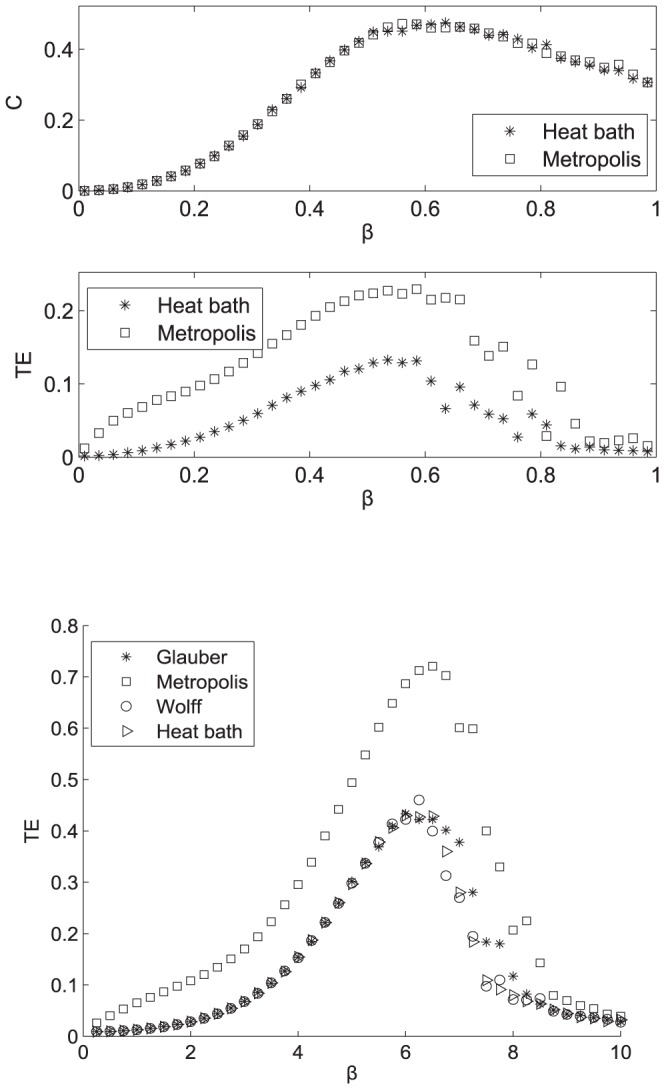
Ising model with different dynamics. a: Transfer Entropy versus the inverse temperature 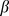 for the Ising model implemented on the 66-nodes human connectome with four different dynamics (Glauber, Metropolis, Wolff and Heat Bath). b: Transfer Entropy versus the inverse temperature 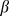 for the Ising model implemented on an deterministic scale free network (81 nodes) with Heat Bath and Metropolis dynamics.

Moreover we stress that the amount of information flow depends on the updating scheme, but the maximum is attained in correspondence of the same coupling, c.f. figure (4a) where four different dynamics (Metropolis, Glauber, Wolff [28] and heat bath) are compared for the 66-nodes brain network. It is interesting to remark that the maximization of information transfer at criticality is not an ubiquitous property of Ising models on networks. Implementation of the same model on a finite size 2D lattice revealed a peak in the paramagnetic phase (figure S1). On the other hand the maximum of information transfer approached the maximum specific heat in a deterministic scale free network [29] (figure 4b). This particular property is thus shared with the brain structural architecture, providing evidence of a family of hierarchical networks supporting it. We remind that the transfer entropy may be seen as a dynamical counterpart of the mutual information, the static measure of statistical dependencies among components of the whole system. It is well known that for a large class of dynamical systems that the mutual information peaks at the order-disorder phase transition [30], [31].

Another interesting remark is that the human connectome has a finite size and inhomogeneous topology. It follows that lowering the temperature, from the paramagnetic phase, the spins will be characterized by different average time between flips 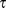. The distribution of 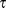 provides, therefore, a further dynamical characterization of the critical state of these Ising system. Close to criticality, the regions for which 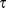 is large may be seen as already ferromagnetic, those with 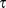 close to one as still paramagnetic: this coexistence may justify a description of the critical dynamics of Ising models on brain in terms of an extended range of temperature, rather than a well-defined critical temperature, thus in line with the broadband criticality studied in the brain in [32] and explained by the theory proposed in [33] describing critical brain behavior in terms of Griffith phases. The regions with large 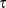 are those driving the rest of the system towards a low-temperature phase or the other as the temperature is further decreased.

**Figure 5:**
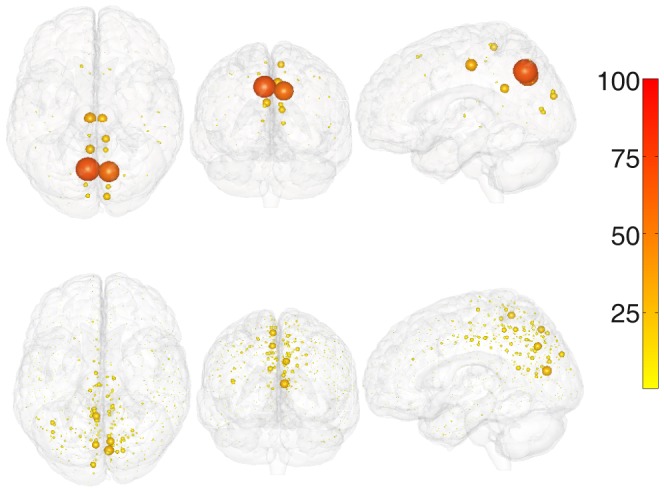
The average time between two consecutive spin flips across the different brain areas. 66-nodes and 998-nodes connectomes.

Figure (5) shows at criticality 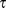 for both the 66-nodes and the 998-nodes connectomes; we note that close to criticality not all the regions appear to be involved in the transition; indeed, most of the regions are displaying a dynamics which is nearly paramagnetic. Thus, for instance, selecting the regions with 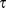 greater than 5, close to criticality and for the 998-nodes connectome, leads to 90 regions having relevant intersections with the DMN, the visual, the somatosensory and the dorsal functional connectivity modules of the resting brain. We remark that both the connectivity and weights of the connectome determine these regions: implementing the Ising model on a network obtained by shuffling the 998-nodes connectome while preserving the degree distribution, the set of regions which are *responsible* of the transition are altered (results not shown). We also note that the pattern corresponding to 998 nodes shows some similarities with the regions with high ROI centrality, ROI strength and network cores of the connectome described in [27].

It is also worth noting that the linear correlation between 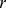 and 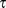, at criticality for the 998 connectome, is 0.3623; this means that 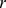 and 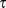 are significantly correlated, although they measure evidence different features of the system dynamics.

**Figure 6:**
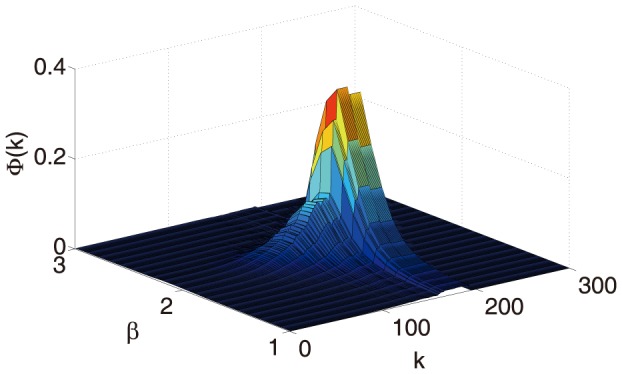
Rich club coefficient 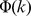 for the network of the information transfer as a function of 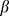 and the degree 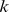.

Finally, we have investigated how the dynamics modulates the tendency of hubs of information flow to be connected to other hubs. This phenomenon, called rich club organization, has been reported in the human brain structural connectome in [34]. This has consequences for the structure of the information flow network. We have found that the rich club coefficient at level k, the fraction of edges that connect nodes of degree k (or higher) out of the maximum number of edges that such nodes might share, has a peak at criticality for at large 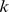, see figure (6) for the 998-nodes connectome, but a similar pattern holds for the 66 nodes connectome (not shown). It follows that the critical state is also characterized as the one such that hubs are maximally rich-club in terms of communications: this is not surprising as at criticality hubs must exchange information in order to change the organization of the system undergoing the phase transition. Since the rich club coefficient does not depend on the strength of connections, we find the same rich club coefficient for the input flows and for the outgoing flows.

## Discussion

The study of the interplay between the brain anatomical network and the neural processes living on it is a challenging topic in neuroscience [35]. In recent studies [17], [18], [36]–[38] spontaneous brain activity has been simulated implementing models of dynamical oscillators with different levels of complexity and biological foundation on the connectome structure, retrieving in some cases correlation-based networks similar to those observed from the analysis of neuroimaging data (mainly fMRI at rest), even with models less biologically realistic such as the Ising one. The present work extends the analysis to dynamical networks who take into account lagged and directional influences using the Ising model, which is easily associated with information theoretical measures, and for which Granger causality is a good approximation to Transfer Entropy. We have shown that the critical state of the Ising model on a brain network is characterized by the maximal amount of information transfer among units, and that brain effective connectivity networks may also be considered in the light of the law of diminishing marginal returns: some units more prominently express this disparity between incoming and outgoing information and are thus liable to become bottlenecks of information. This property of nodes is correlated with both the strength and the average time between spin flips of nodes.

Recently it has been suggested [33] that the modular organization of brain across many scales may be responsible for an extended range of critical brain dynamics, which can be described by Griffiths phases. It is worth mentioning that human brain functional networks have been found to be hierarchically modular[39]. In the present work the Ising model is implemented on the connectome at two different scales, thus providing a study of criticality across scales. We have found that criticality on the connectome at the macro and the mesoscale has the same characteristics. Further investigation are needed to assess if this is peculiar to the connectome architecture or it holds for a generic hierarchical modular network. We suggest that the distribution of weights of the links, coupled to the heterogeneity, is crucial for the connectome in order to show such characterization.

Apart from the insights on how structure and dynamics interact to generate brain function, the approach here described could have more general implications revealing nodes of a network which are particularly representative [40] or influential for the others [41].

## Materials and Methods

The anatomical connectivity matrices used in this study describe the brain at a mesoscale (989 nodes), as well as at a macroscale (66 nodes). They are built as described in [27] from diffusion spectrum imaging and white matter tractography and provided by one of the authors of the original paper. They describe a weighted symmetrical network in which the nodes are normalized cortical regions of interest and the links between them are proportional to the number of connections per unit surface.

The coupling connectivity of the Ising model is given by 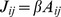, where 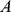 is the 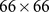 or the 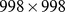 anatomical connectivity matrices, which correspond to undirected weighted networks of human brains, with the weight given by the density of connecting neural fibers. The parameter 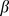 plays the role of an inverse temperature. Whilst in [14] we studied the diluted Ising model on an artificial network, here we analyze the Ising model on the structural architecture of the brain, characterized by two main modules corresponding to the two hemispheres; we estimate the information flow in terms of the total bivariate transfer entropy (summing over all pairs of spins connected by a non-vanishing interaction), which is defined as follows. Let us consider the configurations 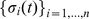 of an Ising system of 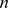 spins living on an arbitrary network. The lagged spin vectors are denoted 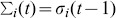. For each pair of spins 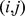 connected by a link in the underlying network, the bivariate transfer entropy 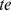, measuring the information flow 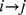 is evaluated as follows: 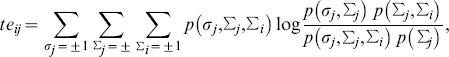
(1)where 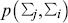 is the fraction of times that the configuration 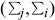 is observed in the data set, and similar definitions hold for the other probabilities. 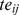 is zero if spins 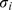 and 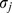 are not connected by a link in the network. We remark that direct evaluation of the multivariate transfer entropy is feasible only for very small systems; a promising approach, which might render larger systems tractable, is described in [42] where transfer entropy is expressed as a likelihood ratio. Moreover, we have also applied the Granger causality analysis to the Ising configurations. We briefly recall the notion of Granger causality, and refer the reader to [43], [44]. Let us consider 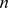 continuous time series 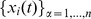; the lagged state vectors are denoted 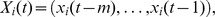


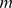
 being the window length. We denote 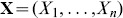 the collection of all variables at hand. Let 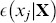 be the mean squared error prediction of 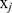 on the basis of all the variables 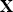, obtained by linear regression. The mean squared error prediction of 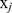 on the basis of all the variables but 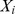, will be denoted 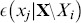. The multivariate Granger causality index 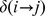 is defined so as to measure the variation of the error in the two conditions, i.e. 
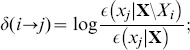
(2)


The formalism of Granger causality is constructed under the hypothesis that time series assume continuous values and are Gaussianly distributed. In the case of binary variables, the Gaussian approximation of the Ising model leads to the approximation 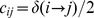, where 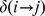 is obtained applying the Granger causality formalism (with 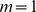) to the to 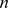 spin time series 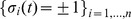, with the substitutions 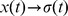 and 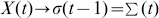 [43]. Applying Granger causality to the Ising model on the brain network, we obtain very similar results to those from bivariate transfer entropy (it has been shown that for Ising models Granger causality provides a good approximation to the transfer entropy while being computationally more efficient [43]). The total information flow is given by 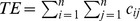. Samples of 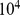 iterations, after discarding transients of 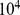 iterations are used to estimate the transfer entropy; we verified that these samples are long enough to provide robust results.

The parameter connected to the law of diminishing marginal returns is 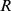, the ratio between the standard deviation 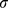 of the distributions of the outgoing information 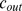 and the incoming information 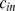 [14], 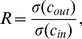
where 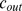 (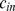) is obtained by summing over columns (rows) the matrix 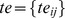 of the information flows 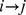 as estimated by bivariate transfer entropy. Moreover, the modulation of the law of diminishing marginal returns is analyzed evaluating, at each node, the ratio between the outgoing and the incoming information: 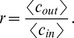



The network measures are defined as in [2] and calculated using the functions connected in the Brain Connectivity Toolbox described there.

## Supporting Information

Figure S1
**Ising model on a 2D lattice.** a: The sum of bivariate transfer entropies for all network pairs is depicted as a function of the inverse temperature 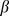 for the 2D Ising model on a square lattice of size 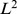, with 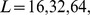 and 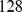. Simulations were performed with a Glauber dynamics and periodic boundary conditions. The vertical line corresponds to the critical point. The curves are shown to converge for L greater than 16. b: The Ising model on a 2D square lattice of size 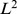, with 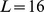. Two different dynamics have been implemented: Metropolis (asterisks) and Glauber dynamics (squares). Although it exists a rescaling in both curves, their shape keep the same. a,b: Transfer entropies have been evaluated averaging over 20 runs of 10000 iterations, from a random initial condition and after stationary state convergence.(PDF)
