# Supplementary material for: Information Transfer and Criticality in the Ising Model on the Human Connectome
Source: PLoS One. 2014 Apr 4;9(4):e93616. doi: 10.1371/journal.pone.0093616 (PMC3976308; doi:10.1371/journal.pone.0093616)
Supplement: Figure S1 — Ising model on a 2D lattice. a: The sum of bivariate transfer entropies for all network pairs is depicted as a function of the inverse temperature for the 2D Ising model on a square lattice of size , with and . Simulations were performed with a Glauber dynamics and periodic boundary conditions. The vertical line corresponds to the critical point. The curves are shown to converge for L greater than 16. b: The Ising model on a 2D square lattice of size , with . Two different dynamics have been implemented: Metropolis (asterisks) and Glauber dynamics (squares). Although it exists a rescaling in both curves, their shape keep the same. a,b: Transfer entropies have been evaluated averaging over 20 runs of 10000 iterations, from a random initial condition and after stationary state convergence. (PDF) [file pone.0093616.s001.pdf]

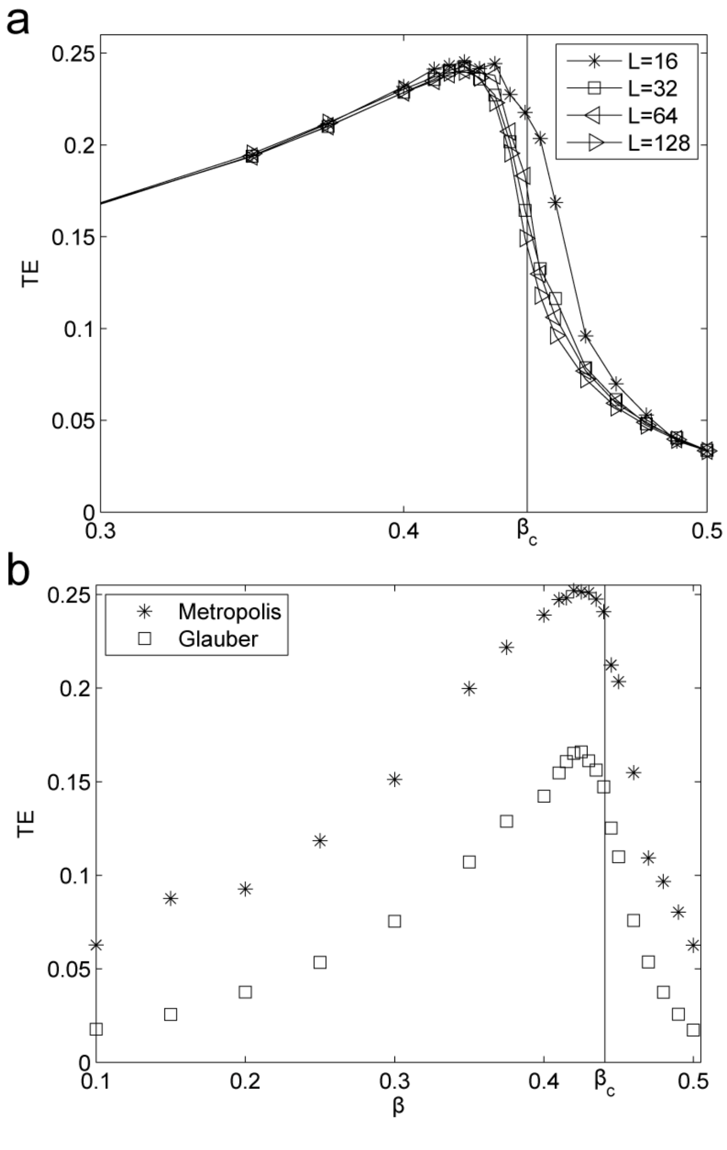

Figure 1: **Ising model on a 2D lattice.** a: The sum of bivariate transfer entropies for all network pairs is depicted as a function of the inverse temperature β for the 2D Ising model on a square lattice of size L^2, with $L = 16, 32, 64$, and 128. Simulations were performed with a Glauber dynamics and periodic boundary conditions. The vertical line corresponds to the critical point. The curves are shown to converge for L greater than 16. b: The Ising model on a 2D square lattice of size L^2, with $L = 16$. Two different dynamics have been implemented: Metropolis (asterisks) and Glauber dynamics (squares). Although it exists a rescaling in both curves, their shape keep the same. a,b: Transfer entropies have been evaluated averaging over 20 runs of 10000 iterations, from a random initial condition and after stationary state convergence.
